# Characterization of an Onboard Transmission Detector for Efficient Linear Accelerator Output Quality Assurance

**DOI:** 10.7759/cureus.48742

**Published:** 2023-11-13

**Authors:** Jeremy Kunz, Vikren Sarkar, Adam B Paxton, Stephen Bhagroo, Geoff Nelson, Bill Salter

**Affiliations:** 1 Radiation Oncology, University of Utah - Huntsman Cancer Institute, Salt Lake City, USA

**Keywords:** delta4 discover, output, efficient, quality assurance, transmission detector

## Abstract

Purpose

To investigate the potential to perform linear accelerator output quality assurance (QA) with the ScandiDos Delta4 Discover (Discover) onboard transmission detector.

Materials and methods

Using the ScandiDos Delta4 software (version 8), a conversion factor from raw signal to output was obtained via cross-calibration with an accredited dosimetry calibration laboratory (ADCL) calibrated ionization chamber for each photon energy, including flattening-filter-free (FFF) energies. With the calibration factor for 6 MV (6x) photon energy, output measurements were taken with both the Delta4 Discover and ion chamber and compared for output as a function of gantry angle and dose-rate dependence. Monitor unit (MU) linearity for 6x was measured and compared with ion chamber measurements. Additionally, the Discover was used to take output measurements, for 6x, approximately every hour throughout the course of a treatment day, and compared with ion chamber output measurements at the beginning and end of the treatment day.

Results

Output measurements for each photon energy were comparable with a maximum difference of -0.57% for flattened beams (6x) and 0.21% for FFF beams (10FFF). Output measurements using the Discover matched ion chamber output measurements at every dose rate within 2%, and within 1% for output as a function of gantry angle. MU linearity test agreed with ion chamber measurements with a maximum difference of 0.41%. Output measurements using the Discover showed a daily drift in output throughout the course of a treatment day of around 2% and correlated very well with ion chamber outputs measured at the beginning and end of the treatment day (within 0.2%).

Conclusions

The ScandiDos Delta4 Discover onboard transmission detector is able to accurately measure linear accelerator output comparable to ion chamber measurements.

## Introduction

One of the medical physicist’s primary responsibilities is to ensure the safety and quality of linear accelerator performance [1-2]. As such, many studies have been dedicated to determining best practices and methods to perform quality assurance (QA) on medical linear accelerators [3-11]. With the increasing complexity of medical linear accelerators and treatment techniques, implementation of a proper QA program can be time-consuming and cumbersome. Recent emphasis has been placed on improving efficiency; allowing for the reallocation of the medical physicist’s time and resources [12-17]. A recent American Association of Physicists in Medicine (AAPM) task group report describes how reallocation of QA resources can be achieved while maintaining a high degree of safety and considering the unique environment at each center [18].

The ScandiDos Delta4 Discover (ScandiDos, Uppsala, Sweden) is a transmission detector consisting of 4040 p-type diodes that affixes directly to the treatment head of the TrueBeam (Varian, Palo Alto, USA) linac via a hook system, similar to the accessory mount. Diodes are spaced every 2.5 mm along the Multi-leaf Collimator (MLC) trajectory and coincide with MLC spacing perpendicularly for a standard collimator TrueBeam machine for a maximum detection area of 20 cm x 25 cm. Although the detector resides on the head of the linac and remains directly in the beam path, it has been shown to have minimal impact on perturbing the treatment beam [19]. Current clinical use of the Discover is for performing patient-specific QA before and during beam delivery as described by Sarkar et al. [20]. In addition to its abilities for radiation detection, the Discover also contains a built-in gyroscope and inclinometer for detecting collimator and gantry angles, respectively. A vendor-independent, onboard transmission detector has the potential to enable efficient detector setup for monitoring a multiplicity of QA requirements. This work seeks to evaluate the potential to perform linear accelerator QA with the ScandiDos Delta4 Discover onboard transmission detector.

## Materials and methods

In order to quantify the output using the Discover, a conversion factor must first be obtained. With the monthly output phantom, consisting of a 20 cm x 20 cm x 30 cm slab of solid water, and an accredited dosimetry calibration laboratory (ADCL) calibrated ion chamber (IC) at 5 cm depth, multiple measurements were taken with the IC, and no Discover in the beam path, and the Discover over a wide range of simulated outputs, from 0.95 cGy/monitor unit (MU) to 105 cGy/MU, for both the IC and Discover, using a dose-rate of 600 MU/min for all energies, with the exception of 10FFF, where 800 MU/min was used. By means of the ScandiDos Delta4 software (version 8), the raw signal from the Discover was exported to a spreadsheet, where the central 5 mm x 5 mm portion of a 10 cm x 10 cm field was averaged for each delivered output mentioned. The IC-measured output was then divided by the average raw signal to obtain an output-per-raw signal factor for each measurement. The output-per-raw signal factors for each delivered output mentioned were averaged to obtain a global calibration factor to convert Discover raw signal to output. This was done for each photon energy, including FFF beams (Table 1).

**Table 1:** Shows global calibration factor for converting Discover raw signal to output for each linear accelerator energy.

Energy (MV)	Discover Conversion Factor (output/raw signal)	Standard Deviation (output/raw signal)
6X	1.18028 x 10^-8^	2.71 x 10^-11^
6FFF	1.08809 x 10^-8^	7.7 x 10^-12^
10X	1.54574 x 10^-8^	5.82 x 10^-11^
10FFF	1.44669 x 10^-8^	1.83 x 10^-11^
18X	1.56879 x 10^-8^	6.86 x 10^-11^

With the calibration factor for each energy, the outputs were measured during a separate session utilizing the same dose rates used to obtain the calibration factor, to test Discover's ability to consistently measure the output of each energy once a conversion factor is obtained. Using the IC in the monthly output phantom, dose-rate dependent output, from 5 MU/min up to 600 MU/min, was measured and compared to Discover measured values by delivering 100 MU at each dose rate to both the IC setup and the Discover. Using the calibration factor obtained for 6x only, output measurements were taken with both the Discover and IC (in a cube of solid water 30 cm x 30 cm x 30 cm) as a function of gantry angle by delivering 100 MU to each set up at the four cardinal gantry angles. MU linearity was measured and compared with ion chamber measurements for 6x photon energy by delivering 2 MU to 800 MU at a dose rate of 600 MU/min. Additionally, the Discover was used to take output measurements approximately every hour throughout the course of a treatment day, and compared with IC output measurements at the beginning and end of the treatment day for 6x, in order to demonstrate linac output variations throughout the course of a typical treatment day.

## Results

**Figure 1:**
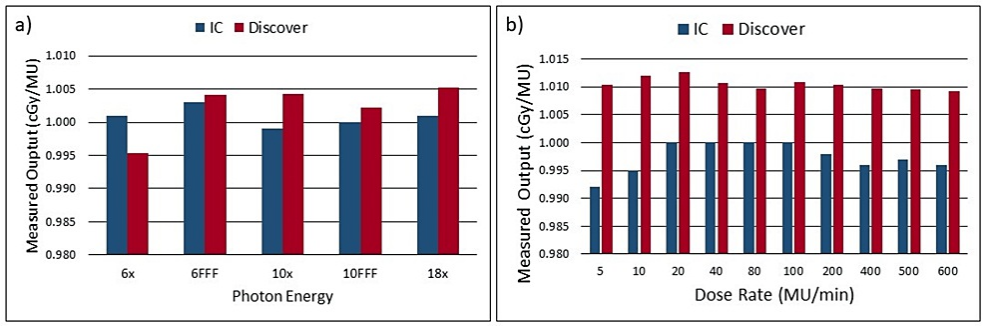
(a) Plot showing IC measured output and Discover measured output for each photon energy, (b) IC measured output and Discover measured output as a dose-rate for 6x photon energy. IC: ion chamber

Output measurements for each photon energy were comparable between IC-measured values and Discover-measured values. The maximum difference observed for flattened beams was -0.57% for 6x, and the maximum difference observed for FFF beams was 0.21% for 10FFF. Figure 1a shows the IC-measured output and Discover-measured output for each photon energy. Figure 1b shows the output measured for both the IC and the Discover at each dose rate. The dose-rate-dependence assessment of the Discover had slightly larger deviations from the IC than previous tests with the smallest being 0.98% at a dose rate of 80 MU/Min and the largest being 1.85% at a dose rate of 5 MU/Min. It is interesting to note that although slightly different from the IC measurement at each dose rate, the Discover showed less fluctuation in measured output at each dose rate than the IC measured output.

**Figure 2:**
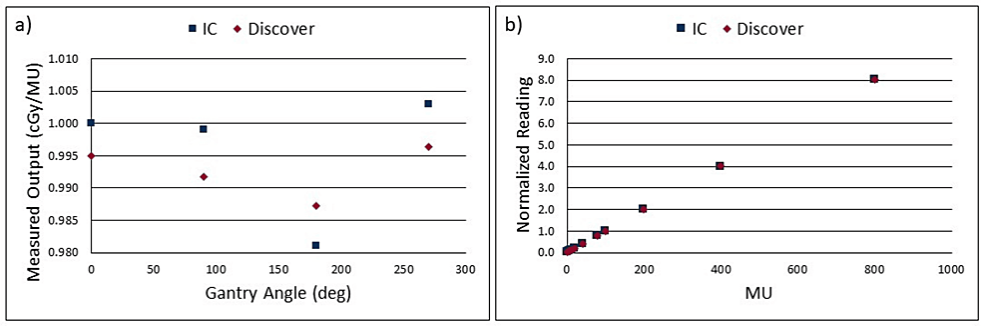
(a)Plot showing IC measured output and Discover measured as a function of gantry angle, (b) IC measured MU linearity and Discover measured MU linearity. Each measurement was performed using 6x photon energy. IC: ion chamber

Figure 2a shows the output measured at the four cardinal gantry angles with both the Discover and an IC. All Discover measured output values were within ±1% of IC measured values. Within the four IC measurements, a maximum difference from the output value at 0 degrees of -1.9% (for 180 degrees) was observed. This discrepancy was most likely due to the tabletop being directly in the beam path; a common concern with this particular test. The maximum difference within the four Discover measurements from the 0-degree output was -0.78% (also for 180 degrees). MU linearity measurements with the IC and the Discover were remarkably similar (Figure 2b). The largest deviation was for 5 MU delivered which showed a difference of 0.41%.

**Figure 3:**
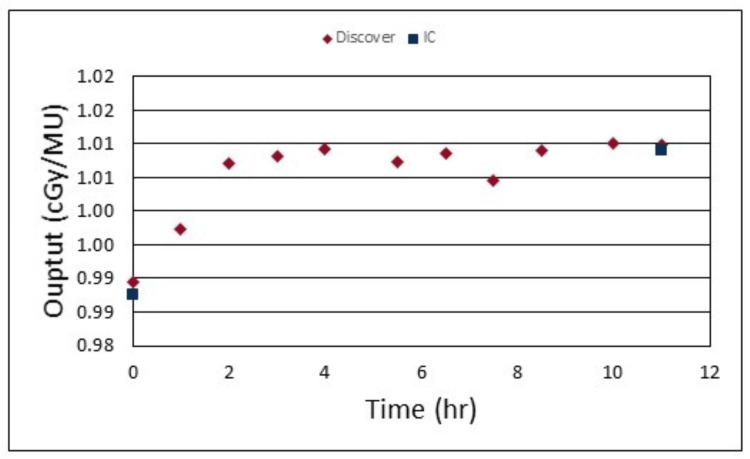
Plot of Discover measured 6x outputs approximately every hour throughout the course of a typical treatment day. Output was also measured at the beginning and end of the treatment day with an IC and compared. IC: ion chamber

Figure 3 shows the output fluctuations measured by the Discover throughout the course of a typical treatment day. The time zero measurement was taken at the time of the therapist’s morning warmup and compared to the IC measurement of the DailyQA3 device (Sun Nuclear, Melbourne, FL). This showed a difference of 0.19% between the Discover measured output and the DailyQA3 measured output, from the central axis IC. The Discover showed a fluctuation in the output of the linear accelerator throughout the course of the treatment day by up to 2.08% from the time zero measurement. This last measurement with the Discover was immediately followed by an IC-measured output using our monthly output QA phantom and showed agreement within 0.09% of the measured outputs.

## Discussion

The Delta4 Discover is able to accurately detect linear accelerator outputs, when compared to IC-measured outputs, for all relevant photon energies. It offers the potential for improved efficiency for performing linear accelerator QA by greatly simplifying detection setup geometry and by its versatility, as it connects directly to the head of the linear accelerator and remains in the beam path throughout treatment. This allows it to efficiently and accurately detect linear accelerator output continuously throughout the day. With an accurate understanding of the temporal changes in linear accelerator output performance, there is potential to influence the medical physicist's determination of when output baselines should be set and adjustments made. Additionally, it is a vendor-independent technique that helps provide an added layer of confidence in the system.

In this work, we explored only a part of Discover’s potential to perform linear accelerator QA; focusing specifically on output. However, there are conceivably many other applications that Discover may be well suited to perform. One such application is for mechanical QA of the collimator and gantry angles due to the built-in gyroscope and inclinometer. Another potential application is that of MLC QA. Sarkar et al. have shown that Discover is able to detect submillimeter deviations in leaf positions in dynamic leaf plans [20]. Therefore, it may be possible to perform regular MLC QA with tests such as the MLC picket-fence test with the Discover.

Limitations to implementing Discover as a viable QA tool exist and must be considered by the user. One such limitation is the upfront time required to obtain an appropriate calibration factor to convert the Discover raw signal to output. This process can be time-consuming on the front end of the implementation. However, this is also true for setting up any output check device. The simplicity of the Discover and the fact that there would be no set-up time, given that it is already on the head of the linac, may mitigate this limitation and be a potential advantage over other output check modalities. The setup for output vs. gantry angle would also be very fast for the same reason and much less cumbersome than using an IC and phantom while attempting to account for the couch at 180 degrees. Another such limitation is that of the analysis process. Currently, only a non-commercial version of the ScandiDos Delta4 software allows the user to obtain the raw signal from Discover’s detectors. Once the raw signal is obtained, the analysis can be cumbersome; requiring the user to manually average the diode signal and convert it to output. We would recommend that the vendor add QA analysis to the software to improve the functionality and efficiency of performing QA with Discover. Additionally, with diode degradation over time being a potential issue, the calibration factor should be checked against IC measurements periodically, and recalibration be performed as needed to ensure a current calibration factor. However, according to the manufacturer's specifications, the long-term sensitivity decrease of the diodes is <0.1%/kGy for 6x.

## Conclusions

In this work, we measure and report the linear accelerator output using the ScandiDos Delta4 Discover and compare it to IC-measured output. The Discover is able to accurately detect linear accelerator output for all relevant photon energies while greatly simplifying setup geometry and uncertainties. Potential for other QA applications for Discover also exists to further improve efficiency.
